# Changes in arterial pressure and markers of nitric oxide homeostasis and oxidative stress following surgical correction of hydronephrosis in children

**DOI:** 10.1007/s00467-017-3848-4

**Published:** 2017-12-01

**Authors:** Ammar Al-Mashhadi, Antonio Checa, Nils Wåhlin, Tryggve Neveus, Magdalena Fossum, Craig E. Wheelock, Birgitta Karanikas, Arne Stenberg, A. Erik G. Persson, Mattias Carlstrom

**Affiliations:** 1 Pediatric Surgery Section, Department of Women’s and Children’s Health, Uppsala University, Uppsala, Sweden; 2 Department of Medical Biochemistry and Biophysics, Karolinska Institutet, Stockholm, Sweden; 3 Department of Women’s and Children’s Health, and Center for Molecular Medicine, Karolinska Institutet, Stockholm, Sweden; 4 Department of Pediatric Surgery, Astrid Lindgren Children’s Hospital, Karolinska University Hospital, Stockholm, Sweden; 5 Pediatric Nephrology Unit, Department of Women’s and Children’s Health, Uppsala University, Uppsala, Sweden; 6 Department Medical Cell Biology, Uppsala University, Uppsala, Sweden; 7 Department of Physiology and Pharmacology, Karolinska Institutet, Nanna Svartz Väg 2, 17177 Stockholm, Sweden

**Keywords:** Blood pressure, Hydronephrosis, Hypertension, Nitric oxide, Oxidative stress, Ureteral obstruction

## Abstract

**Objective:**

Recent clinical studies have suggested an increased risk of elevated arterial pressure in patients with hydronephrosis. Animals with experimentally induced hydronephrosis develop hypertension, which is correlated to the degree of obstruction and increased oxidative stress. In this prospective study we investigated changes in arterial pressure, oxidative stress, and nitric oxide (NO) homeostasis following correction of hydronephrosis.

**Methods:**

Ambulatory arterial pressure (24 h) was monitored in pediatric patients with hydronephrosis (*n* = 15) before and after surgical correction, and the measurements were compared with arterial pressure measurements in two control groups, i.e. healthy controls (*n* = 8) and operated controls (*n* = 8). Markers of oxidative stress and NO homeostasis were analyzed in matched urine and plasma samples.

**Results:**

The preoperative mean arterial pressure was significantly higher in hydronephrotic patients [83 mmHg; 95% confidence interval (CI) 80–88 mmHg] than in healthy controls (74 mmHg; 95% CI 68–80 mmHg; *p* < 0.05), and surgical correction of ureteral obstruction reduced arterial pressure (76 mmHg; 95% CI 74–79 mmHg; *p* < 0.05). Markers of oxidative stress (i.e., 11-dehydroTXB_2_, PGF_2α_, 8-*iso*-PGF_2α_, 8,12-*iso*-iPF_2α_-VI) were significantly increased (*p* < 0.05) in patients with hydronephrosis compared with both control groups, and these were reduced following surgery (*p* < 0.05). Interestingly, there was a trend for increased NO synthase activity and signaling in hydronephrosis, which may indicate compensatory mechanism(s).

**Conclusion:**

This study demonstrates increased arterial pressure and oxidative stress in children with hydronephrosis compared with healthy controls, which can be restored to normal levels by surgical correction of the obstruction. Once reference data on ambulatory blood pressure in this young age group become available, we hope cut-off values can be defined for deciding whether or not to correct hydronephrosis surgically.

## Introduction

The kidneys play a key role in whole body fluid and electrolyte homeostasis and hence in long-term regulation of the arterial pressure. Abnormal renal autoregulation of glomerular perfusion and filtration as well as intrinsic renal disease can either cause hypertension or be a consequence of this condition [1]. Obstruction at the level of the pelvo-ureteric junction and subsequent development of hydronephrosis is a fairly common condition in newborns, with an incidence of approximately 1% [2]. Currently there is limited clinical knowledge regarding the influence of hydronephrosis on arterial pressure regulation. Although hypertensive effects of hydronephrosis have been suggested in experimental studies and clinical case reports [3–5], this has not been substantiated by prospective studies in humans. It has been shown that the function of the hydronephrotic kidney in many cases remains surprisingly well preserved for several years [6, 7]. This observation has lead to a worldwide trend towards non-operative treatment, but the long-term effects on cardiovascular and renal function of this treatment policy are not known [2].

Recently, we showed for the first time in a prospective case study that surgical relief of hydronephrosis, secondary to ureteral obstruction in children, is associated with a lowering of arterial pressure [8]. Moreover, other studies have shown that both rats and mice with chronic partial unilateral ureteral obstruction (PUUO) or congenital hydronephrosis develop hypertension [9–11]. In previous experimental studies we demonstrated that hypertension and salt-sensitivity significantly correlated with the degree of hydronephrosis and that the disease was associated with oxidative stress and nitric oxide (NO) deficiency in the diseased kidney [12–15]. Finally, surgical relief of the obstruction in rats with hydronephrosis significantly attenuated arterial pressure [16].

In this prospective study, our aim was to further study ambulatory arterial pressure (24 h) and different markers of NO homeostasis and oxidative stress in blood or urine in pediatric patients with congenital hydronephrosis before and after surgical correction of the ureteral obstruction. Specifically, we investigated the hypothesis that preoperative arterial pressure is higher in children with hydronephrosis, which can be reduced by surgical management, and that this reduction in arterial pressure would be associated with attenuated oxidative stress and normalized NO homeostasis.

## Research design and methods

### Study population

Fifteen patients with unilateral congenital hydronephrosis together with two age- and sex-matched control groups, i.e., healthy controls (HC, *n* = 8) and operated controls (OC, *n* = 8) were included in this prospective study. The subjects were recruited between 2007 and 2016 at the Pediatric Surgery Department of Uppsala University Children’s Hospital in Uppsala and the Pediatric Surgery Department of Astrid Lindgren’s Hospital in Stockholm (Table 1). We included in this study only children with unilateral hydronephrosis that was not associated with any other disease and who had no other risk factors for hypertension and no prior antihypertensive treatment. Nine patients had left side hydronephrosis while six patients had hydronephrosis on the right side. Seven patients had laparoscopic surgery with pyeloplasty, while eight patients underwent open pyeloplasty. Indications for performing pyeloplasty were significant increases in the anteroposterior diameter of the renal pelvis of the hydronephrotic kidney, decreased function on renography, repeated urinary tract infections, and recurrent abdominal colic.

**Table 1:** Arterial pressure and preoperative radiological examinations in the hydronephrotic group

Age at time of surgery (years)	Operation	AP_DAY_ (Pre)	AP_NIGHT_ (Pre)	AP_DAY_ (Post)	AP_NIGHT_ (Post)	MAG3 (Pre)	RPAD (Pre)	Sex
1	Open pyeloplasty Left (Lt)	108/86	112/76	103/66	93/55	–	–	Male
3^a^	Laparoscopic pyeloplasty Lt	111/65	98/54	105/65	98/54	47% IIIb Lt	18 mm	Male
4^a^	Open pyeloplasty Right (Rt)	116/72	90/54	101/56	92/50	46% II Rt	26 mm	Male
5	Open pyeloplasty Rt	129/76	104/64	121/75	104/57	34% IIIb Rt	22 mm	Male
5	Open pyeloplasty Lt	100/85	100/65	100/85	93/62	42% II Lt	45 mm	Male
6	Open pyeloplasty Lt	107/67	97/60	104/64	100/59	45% II Lt	37 mm	Male
6^a^	Laparoscopic pyeloplasty Lt	105/67	98/66	105/62	92/48	50% II Lt	15 mm	Male
7^a^	Laparoscopic pyeloplasty Rt	119/77	102/52	110/61	102/54	35% IIIb Rt	25 mm	Male
9^a^	Laparoscopic pyeloplasty Lt	115/67	95/61	112/68	101/60	55% II Lt	40 mm	Male
11	Open pyeloplasty Lt	108/68	99/59	103/66	94/56	27% IIIb Lt	15 mm	Male
11	Open pyeloplasty Rt	120/79	106/67	113/71	105/60	47% II Rt	35 mm	Male
11^a^	Laparoscopic pyeloplasty Rt	116/65	108/58	115/72	104/53	49% II Rt	15 mm	Male
12^a^	Laparoscopic pyeloplasty Lt	116/72	101/61	124/71	113/59	49% II Lt	29 mm	Female
12^a^	Laparoscopic pyeloplasty Lt	124/73	116/62	121/71	104/57	50% IIIb Lt	40 mm	Male
12	Open pyeloplasty Rt	154/88	138/75	114/58	110/58	11%/ II Rt	35 mm	Male

AP_DAY_ (Pre), Average arterial pressure (systolic/diastolic values) daytime preoperatively; AP_NIGHT_ (Pre), average arterial pressure (systolic/diastolic values) nighttime preoperatively; AP_DAY_ (Post), average arterial pressure (systolic/diastolic values) daytime postoperatively; AP_NIGHT_ (Post), average arterial pressure (systolic/diastolic values) nighttime postoperatively; MAG3 (Pre), preoperative evaluation of bilateral renal function by mercaptoacetyltriglycine (MAG3) scintigraphy; RPAP (Pre), renal pelvis anterioposterior diameter according to the preoperative ultrasound

^a^Urine and plasma samples were collected before and 6 months after surgery

The control group consisted of eight completely healthy children who did not have any general anesthesia or surgery. Another control group of age-and sex-matched children (*n* = 8) was also included who had undergone general anesthesia for a minor day ward operation, but were otherwise considered to be healthy (Table 2). Arterial pressure data from a subpopulation of hydronephrotic patients were partly communicated in a previous case report [8], but these data were reanalyzed before being included in the current study. Children’s age in all three groups ranged from infancy to 12 years. There were 14 boys and one girl in the hydronephrotic group and eight children in each of the two different control groups.

**Table 2:** Arterial pressure in the healthy and operated control groups

Age (years)^a^	Operation	AP_DAY_	AP_NIGHT_	Sex
3	Healthy control (No surgery)	95/62	85/52	Male
4	Healthy control (No surgery)	111/70	96/57	Male
5	Healthy control (No surgery)	97/61	90/51	Female
5	Healthy control (No surgery)	95/54	89/44	Male
7	Healthy control (No surgery)	109/63	107/58	Male
7	Healthy control (No surgery)	110/69	105/62	Male
10	Healthy control (No surgery)	113/73	114/63	Female
12	Healthy control (No surgery)	107/69	111/64	Male
1	Operated control (Circumcision)	98/61	89/52	Male
2	Operated control (Inguinal hernia)	111/65	124/65	Male
5	Operated control (Circumcision)	107/68	97/60	Male
7	Operated control (Inguinal hernia)	107/67	99/60	Male
7	Operated control (Hydrocele)	130/86	109/61	Male
8	Operated control (Foreskin plastic)	102/60	99/53	Male
10	Operated control (Foreskin plastic)	126/77	100/53	Male
12	Operated control Colonoscopy	120/72	104/57	Female

AP_DAY_, AP_NIGHT_, see footnote of Table 1

^a^Age of patient age in years at time of arterial pressure recording and sample collection

Ambulatory arterial pressure was monitored for 20–24 h, including the full nocturnal period, preoperatively and again 6 months following management of the obstruction in the hydronephrotic group. Similar to the procedure described in previous studies [17, 18], the infant cuff was attached to the non-dominant arm to reduce movement-mediated measurement errors. The monitor was placed in a padded backpack so as not to disturb the infant. We did also ambulatory arterial pressure measurements for the healthy controls and the operated control group (preoperatively), using exactly the same method, device and timing as for the hydronephrotic group (i.e., 20–24 h before surgery). Ambulatory arterial pressure readings were obtained every 20 min during the daytime (0600–2200 hours) and every hour during the nighttime (2200–0600 hours). The same consultant pediatric nephrologist evaluated both preoperative and postoperative blood pressure curves for all patients and both control groups.

In eight subjects from each of the control groups we collected 5–10 ml blood and 10 ml urine for later biochemical analyses (described in following sections). In the hydronephrotic group matched samples were obtained before and 6 months after surgery when arterial pressure measurements were conducted. In the hydronephrotic group, anteroposterior diameter of the renal pelvis was measured by ultrasound a few weeks to a few days preoperatively to confirm the diagnosis of hydronephrosis. Preoperative evaluation of bilateral renal function was performed by mercaptoacetyltriglycine (MAG3) scintigraphy. Tc-99 m-labeled MAG3 renography with forced diuresis was also performed in all patients. Following intravenous injection of the tracer (1 MBq/kg body weight), registration (eCAM gamma camera; Siemens AG, Munich, Germany) continued for at least 30 min, and furosemide (0.5 mg/kg body weight) was given intravenously after 15 min. The renography curves were classified according to O’Reilly [19] as normal (I), obstructed (II), dilated non-obstructed (IIIa), or equivocal (IIIb). Both a pediatric nephrologist and a nuclear medicine specialist assessed the split renal function.

### Analyses of blood and urine samples

Blood samples were immediately centrifuged (4700 *g*, 5 min, 4 °C), and collected aliquots of both plasma and urine were stored at − 80 °C for later analyses of markers of NO homeostasis and oxidative stress, as described in detail in the following sections.

#### Nitrate and nitrite

A high-performance liquid chromatography (HPLC) system dedicated to the assessment of nitrite and nitrate (ENO-20; EiCom, Kyoto, Japan) equipped with an auto-sampler (840; EiCom) were used [20]. The method is sensitive and specific to nitrite and nitrate and is based on the separation of nitrate by reverse-phase/ion exchange chromatography, followed by inline reduction of nitrate to nitrite with cadmium and reduced copper. Derivatization of reduced nitrate was performed with Griess reagent, and the level of diazo compounds was measured at 540 nm. The reactor solution was freshly prepared before analysis. A standard curve was prepared from sodium nitrite and sodium nitrate and diluted with carrier solution, and aliquots of standards were stored at − 20 °C. The slope was examined and used to calculate the concentration in the samples. The plasma samples were deproteinized using HPLC grade methanol (CHROMASOLV Solvent; Sigma-Aldrich, St. Louis, MO). Ice-cold methanol (100 μl) was mixed with plasma (100 μl) in 1.5-ml Eppendorf tubes to test for nitrite/nitrate contamination. The tubes were vortexed and then centrifuged for 10 min (4 °C, 10,000 g), the denaturized proteins thereby forming a pellet. Immediately before running the samples in the HPLC, supernatant (100 μl) was transferred to a 96-well plate with conical wells (Costar, nitrate- and nitrite-free; Corning Inc., Corning, NY), and the plate was sealed with a film and placed in the autosampler. The samples were kept at 4 °C by a cooling device in the autosampler. Aliquots of 10 μl of background control, standard, or samples were injected, and the needle was automatically flushed between each injection by the autosampler.

#### Cyclic guanosine monophosphate

An ELISA kit was purchased from GE Healthcare (Uppsala, Sweden) and run according to the manufacturer’s instructions. Plasma was collected in tubes containing 3-isobutyl-1-methylxanthine (IBMX; 10 μmol/L) to prevent degradation of cyclic guanosine monophosphate (cGMP).

#### Amino acids

Plasma levels of arginine, citrulline, ornithine, and asymmetric and symmetric dimethylarginine (ADMA and SDMA, respectively) were measured by HPLC tandem mass spectrometry (LC-MS/MS) as previously described, with minor modifications [21, 22]. Briefly, after thawing samples at 4 °C, 25 μl of plasma was crashed with 225 μl of 0.2% formic acid in isopropanol containing the internal standard (N4-arginine). The samples were then vortexed for 30 s and centrifuged at 10,000 *g* for 10 min. Finally, 5 μl of the supernatant was injected into the LC-MS/MS system. Separation was performed with an ACQUITY UPLC System from Waters Corporation (Milford, MA) using an Atlantis HILIC Silica 3 μm (150 × 2.1 mm) column from Waters Corporation. Mobile phases consisted of 10 mM ammonium formate + 0.2% formic acid in acetonitrile:methanol (75:25) and 10 mM ammonium formate + 0.2% formic acid in water. The flow rate was set at 400 μl/min. Detection was performed using a Waters Xevo® TQ triple quadrupole equipped with an electrospray ion source working in positive mode. Laboratory plasma reference material was used as a quality control (QC) to ensure reproducibility of quantitation within the study. The reproducibility according to the QCs [(% coefficient of variance (CV) < 8] was within the accepted range (%CV < 15) for all reported compounds.

#### Creatinine

Creatinine was quantified in 10 μl of urine as previously described [23]. Laboratory urine reference material was used as a QC. The reproducibility according to the QCs (%CV = 1.1) for creatinine was within the accepted range (%CV < 15).

#### Oxidative stress

Isoprostanes were quantified in 300 μl of urine using an urinary eicosanoid LC-MS/MS platform as previously described [24]. Three different isoprostane species were quantified, i.e., 8,12-*iso*-iPF_2α_-VI, 8-isoprostane (8-*iso*-PGF_2α_), and its excreted urinary metabolite, 2,3-dinor-8-*iso*-PGF_2α_. Three additional urinary eicosanoids (PGF_2α_, PGE_2_, and 11-dehydroTXB_2_) were also quantified. Fortified laboratory urine reference material was used as a QC to ensure reproducibility of quantitation within the study. The reproducibility according to the QCs (%CV <12) was within the accepted range (%CV < 15) for all reported compounds.

### Statistical analysis

Statistical analyses were performed using GraphPad Prism 6 for Mac OS X (version 6.0b; GraphPad Software Inc., San Diego, CA). The comparisons of the arterial pressure and markers in urine and plasma among groups were analyzed by using analysis of variance (non-parametric Kruskal–Wallis test) followed by Dunn’s multiple comparisons test. Wilcoxon matched-pairs signed rank test (two-tailed) was used to analyze the effect of surgical management on the hydronephrotic group. Data are shown as a box and whiskers (5–95 percentile) graph. Linear regression analysis and the Pearson *r* correlation was used to test for the association between MAG3 and mean arterial pressure. Statistical significance was defined as *p* < 0.05.

## Results

### Arterial pressure

**Fig. 1:**
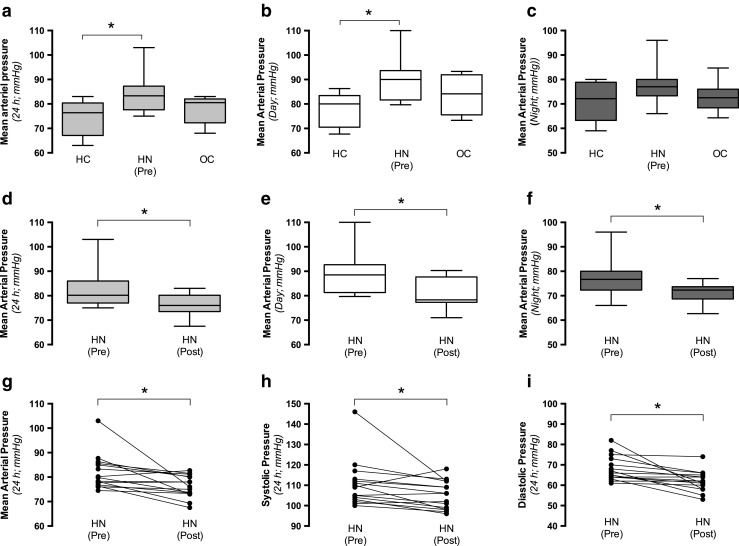
Arterial pressure in controls and hydronephrotic (HN) children. **a**–**c** Mean arterial pressure (MAP) in two control groups [healthy controls (HC) and operated controls (OC)] and HN children before surgical management of the obstruction [HN (Pre)]. MAP is presented for 24 h (**a**), during daytime (**b**), and during nighttime (**c**). **d**–**f** MAP in HN children before [HN (Pre)] and after [HN (Post)] surgical management of the obstruction. Matched arterial pressure data are presented for 24 h (**d**), during daytime (**e**), and during night (**f**). Data in **a**–**f** are presented as box-plots with the median and first and third quartiles (box) and whiskers (minimum to maximum). **g**, **h** Systolic (**g**) and diastolic (**h**) pressures (24 h) in HN before [HN (Pre)] and after [HN (Post)] surgical management of the obstruction. HC, *n* = 8; OC, *n* = 8; HN (Pre), *n* = 15, HN (Post), *n* = 15. Asterisk denotes significance at *p* < 0.05

The 24-h mean arterial pressure (MAP) was significantly higher in the hydronephrotic group than in the healthy control group (Fig. 1a–c), but not significantly (*p* = 0.08) higher than that in the operated control group (preoperatively). Moreover, there was no difference in arterial pressure between the two control groups. Surgical correction of the hydronephrosis was associated with significantly lower arterial pressure (Fig. 1d–f). Postoperatively, there were no differences in arterial pressure among the investigated groups: Healthy controls 74 mmHg [(95% confidence interval (CI) 69–80 mmHg]; operated controls 78 mmHg (95% CI 73–83 mmHg); hydronephrotic group (Post) 76 mmHg (95% CI, 74–79 mmHg). Both systolic and diastolic pressures were significantly reduced following surgical management of the obstruction in children with hydronephrosis (Fig. 1g-h).

### Renal ultrasound

Anteroposterior diameter preoperatively ranged from 15 to 50 mm in the hydronephrotic kidney (Table 1).

### Renal function

**Fig. 2:**
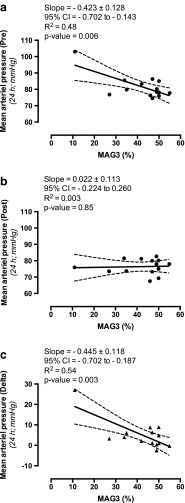
Correlation between mercaptoacetyltriglycine (MAG3) scintigraphy results and MAP. **a**, **b** There was a significant and strong negative linear relationship (*r* = − 0.69) between MAG3 (%) and MAP (24 h) before surgical management of the hydronephrosis (**a**), whereas no correlation (*r* = 0.05) was found at 6 months after surgery (**b**). **c** A significant and strong negative linear relationship (*r* = −0.74) was also found between MAG3 (%) and the degree of blood pressure reduction following surgery. Data are presented as mean and error with 95% CI

The left kidney was more often hydronephrotic than the right (nine vs. five patients; 60 vs. 40%). The renal functional share of the hydronephrotic kidney ranged from 11 to 55% (Table 1). Nine patients had hydronephrosis grade II (obstructed) while five patients had grade IIIb (equivocal). A significant and strong negative correlation was found between MAG3 (%) and MAP (24 h) before surgical management of the hydronephrosis (Fig. 2a), but not 6 months after surgery (Fig. 2b). A significant and strong negative correlation was also found between MAG3 (%) and the change in blood pressure following surgery (Fig. 2c).

### Markers in blood and urine

Several different markers of NO homeostasis and oxidative stress level were measured in the plasma and in urine before and after surgical correction of hydronephrosis in children.

#### Nitrite, nitrate and cGMP

**Fig. 3:**
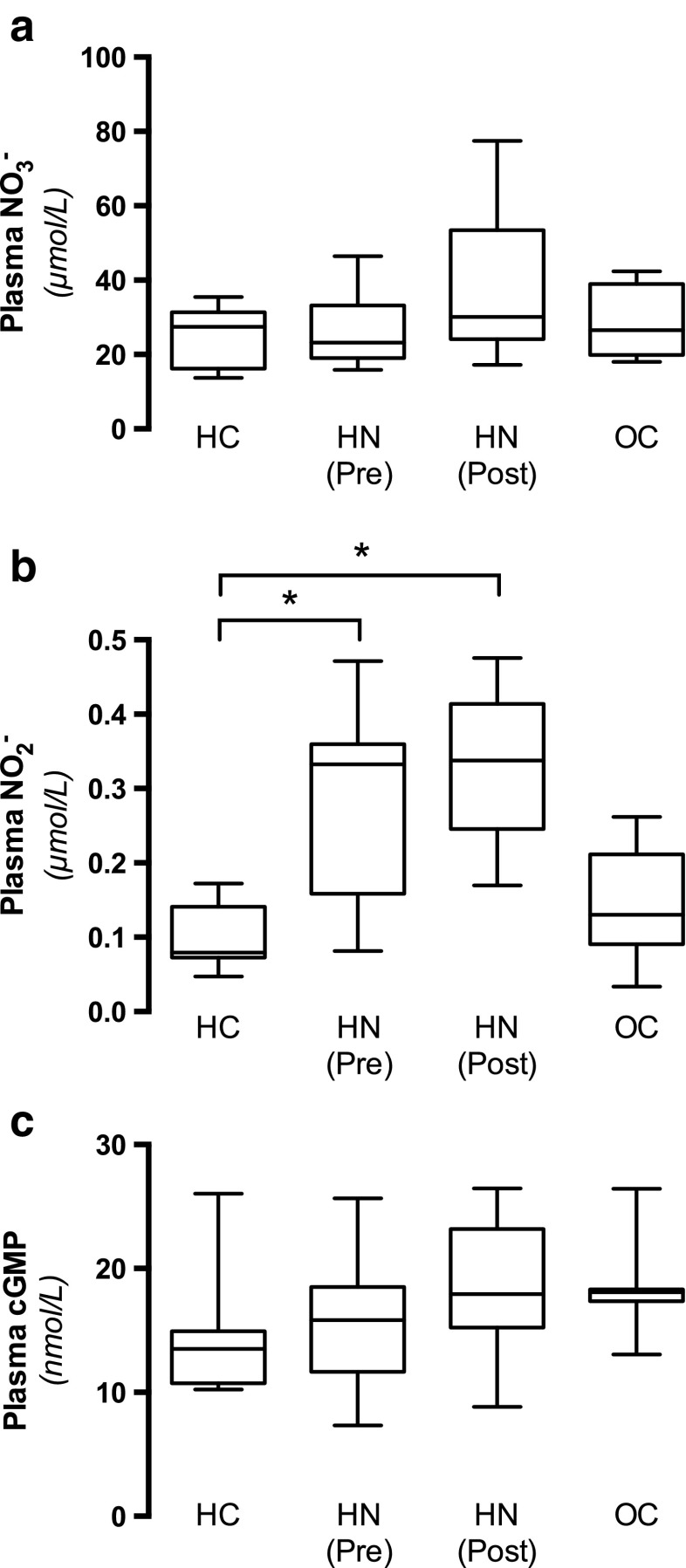
Markers of nitric oxide (NO) homeostasis. Inorganic nitrate (NO_3_^−^; **a**) and nitrite (NO_2_^−^; **b**) as well as a marker of NO signaling [cyclic guanosine monophosphate (cGMP); **c**] was measured in plasma from HC (*n* = 8), OC (*n* = 8), and HN children before [HN (Pre), *n* = 8] and after [HN (Post), *n* = 8] surgical management of the obstruction. Data are presented as box-plots, with the median and first and third quartiles (box) and whiskers (minimum to maximum). Asterisk denotes significance at *p* < 0.05

Nitric oxide can be oxidized to nitrite (NO_2_^−^) and nitrate (NO_3_^−^), and hence the levels of these inorganic anions have been widely used as an index of NO generation. No differences were observed for plasma nitrate (Fig. 3a), whereas plasma nitrite levels were significantly higher in patients with hydronephrosis before and after surgical management compared with controls (Fig. 3b). Moreover, there were no differences in NO signaling among the groups, as indicated by similar plasma levels of cGMP (Fig. 3c). Taken together these results suggest that overall NO homeostasis is not impaired in patients with hydronephrosis and that, to the contrary, the activity of NO generating systems may be increased.

#### Amino acids

**Fig. 4:**
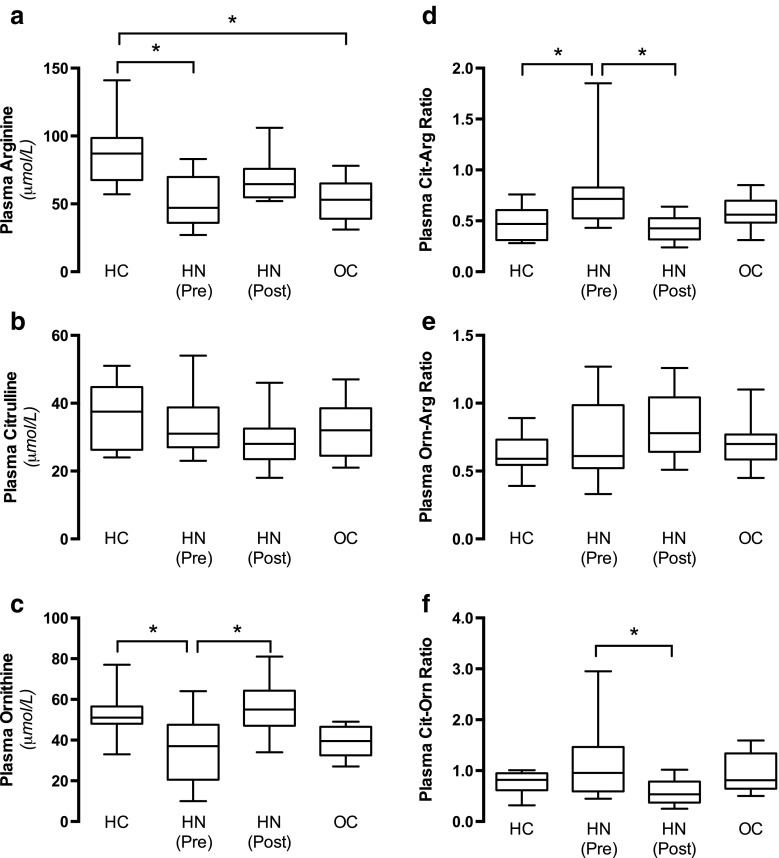
Nitric oxide synthase (NOS) and arginase activity. Substrate for NOS and arginase activity [i.e., arginine (Arg)], and the metabolites from their enzyme activity [citrulline (Clt) and ornithine (Orn), respectively] were measured in plasma (**a**–**c**) from HC ( *n* = 8), OC ( *n* = 8), and HN children before [HN (Pre), *n* = 8] and after [HN (Post), *n* = 8] surgical management of the obstruction. **d**, **e** The citrulline-to-arginine ratio (**d**) and ornithine-to-arginine ratio (**e**) are used as indexes of NOS activity and arginase activity, respectively. **f** The ratio between citrulline and ornithine is used as a relative index of the NOS-to-arginase activity F). Data are presented as median with first and third quartiles (box) and whiskers (minimum to maximum). Asterisk denotes significance at *p* < 0.05

Analyses of plasma arginine, citrulline, and ornithine were conducted, and the ratio for citrulline/arginine [nitric oxide synthase (NOS) activity], ornithine/arginine (arginase activity), and citrulline/ornithine (relative NOS vs. arginase activity) were calculated (Fig. 4a–f). Taken together our results suggest increased NOS activity in patients with hydronephrosis, which is normalized following surgical management (Fig. 4d).

**Fig. 5:**
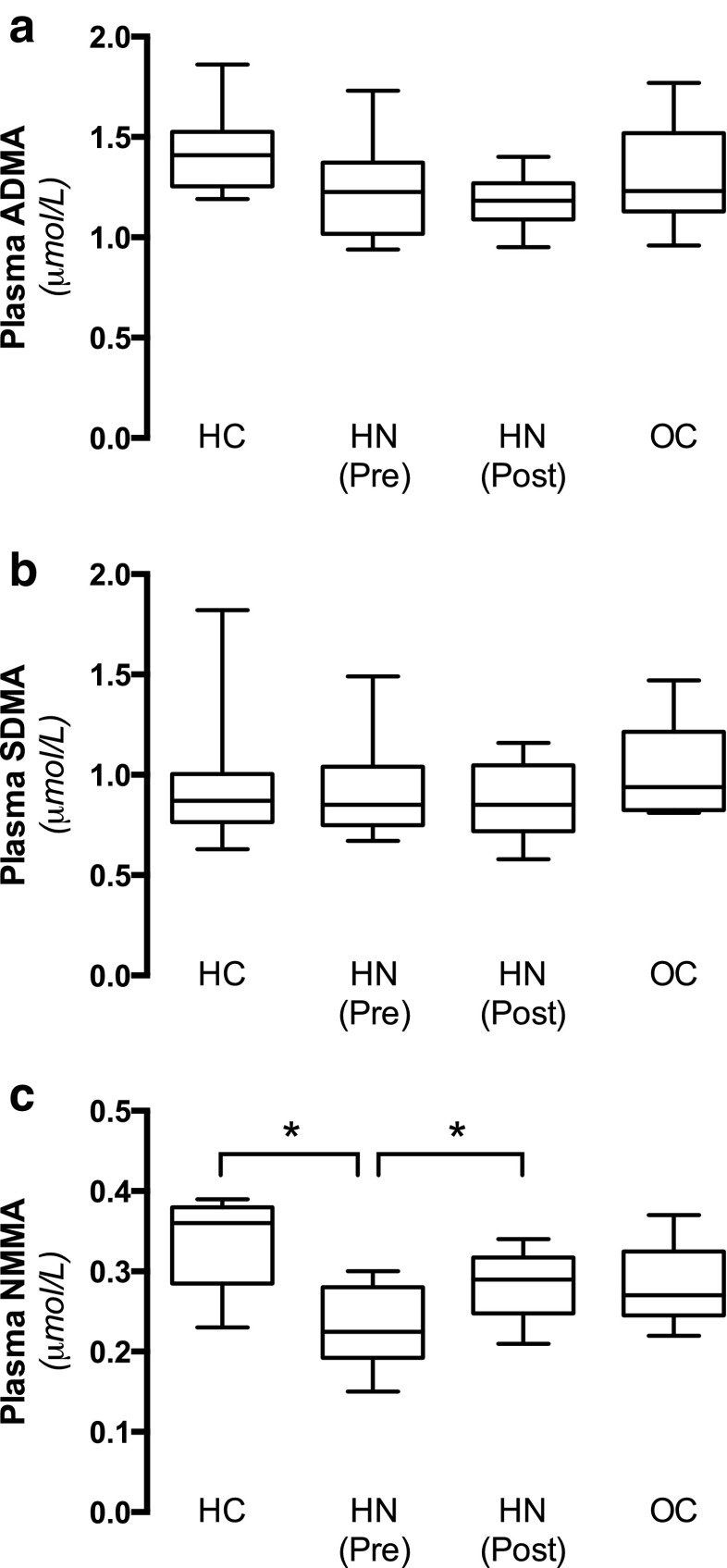
Endogenous inhibitors of NOS activity. Plasma levels of the endogenous NOS inhibitors asymmetric dimethylarginine (ADMA), symmetric dimethylarginine (SDMA), and *N*-monomethyl arginine (NMMA) were analyzed in plasma from HC (*n* = 8), OC ( *n* = 8), and HN children before [HN (Pre), *n* =  8] and after [HN (Post), *n* = 8] surgical management of the obstruction. Data are presented as box-plots, with the median and first and third quartiles (box) and whiskers (minimum to maximum). Asterisk denotes significance at *p* < 0.05

Previous experimental and clinical studies have shown that renal disease and associated hypertension is associated with abnormal regulation of the endogenous NOS inhibitors ADMA, SDMA, and *N*-monomethyl arginine (NMMA) [21, 25, 26]. We did not observe any differences in ADMA or SDMA among the groups (Fig. 5a, b). However, in support of the suggested increased NOS activity data, the levels of the endogenous NOS inhibitor NMMA were significantly lower in hydronephrotic patients compared with the healthy controls, and these levels were normalized following management of the obstruction (Fig. 5c).

#### Oxidative stress

**Fig. 6:**
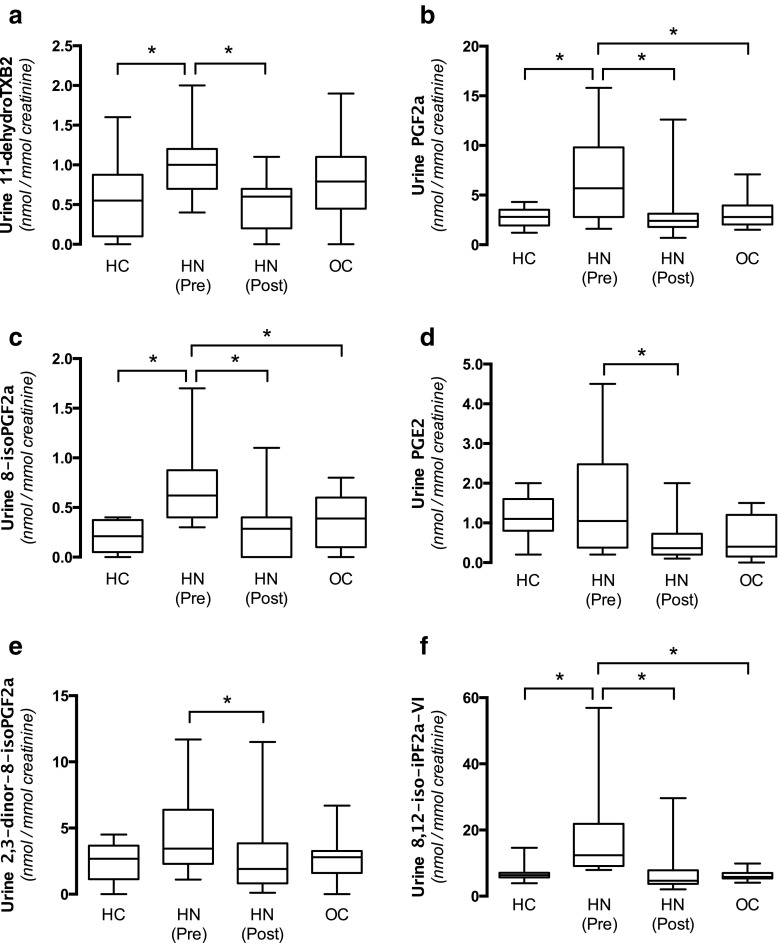
Pathways and markers associated with oxidative stress. Urine markers of prostaglandin and thromboxane signaling that have been associated with oxidative stress (**a**, **b**, **c**, **d**) as well as markers of oxidative stress (**e**, **f**). Urine samples were obtained from HC ( *n* = 8), OC ( *n* = 8), and HN children before [HN (Pre), *n* =  8] and after [HN (Post), *n* =  8] surgical management of the obstruction and the levels were corrected by creatinine. Data are presented as box-plots, with the median and first and third quartiles (box) and whiskers (minimum to maximum). Asterisk denotes *p* < 0.05

We have previously demonstrated that hypertension in rats and mice with experimentally induced hydronephrosis was associated with oxidative stress. In this clinical study we analyzed several lipid markers associated with oxidative stress in urine samples before and after surgical management that were normalized to the creatinine levels. Four markers (11-dehydroTXB_2_, PGF_2α_, 8-*iso*-PGF_2α_, 8,12-*iso*-iPF_2α_-VI) were significantly elevated in hydronephrotic patients compared with healthy controls and these were normalized following surgery (Fig. 6a–c, f). In addition, both PGE_2_ and 2,3-dinor-8-*iso*-PGF_2α_ were significantly lowered following surgical correction of the obstruction (Fig. 6d, e).

## Discussion

The results of the current prospective study are in agreement with those of our previous experimental [9, 10, 16] and clinical studies [8], demonstrating that hydronephrosis in pediatric patients is associated with increased arterial pressure, which can be reduced by surgical relief of the obstruction. As previously shown in experimental rodent models of hydronephrosis [12–15], using partial ureteral obstruction, we show for the first time that also patients with hydronephrosis display elevated levels of markers associated with oxidative stress, which can be reduced following management of the obstruction.

There are currently only two clinical studies in children with hydronephrosis that have discussed the effects of surgical treatment on arterial pressure. The first study by de Waard et al. was a retrospective study that suggested a reduction of arterial pressure after surgical management of dilated or obstructed upper urinary tracts [4]. The second study, conducted by our research group, was a small prospective study demonstrating that systolic and diastolic pressures were reduced following surgical correction of hydronephrosis in children with pelvic ureteric obstruction [8]. However, the latter study did not include a matched control group and the mechanisms were not further investigated. In the current study, we included two age- and sex-matched control groups in order to strengthen our hypothesis of increased arterial pressure in hydronephrosis and its reduction after surgical treatment. The first group consisted of healthy children, but we also included another control group of healthy children who had undergone 24-h ambulatory arterial pressure monitoring before undergoing small surgery for other reasons. We found no difference in arterial pressure between the healthy and operated control groups. Based on our results we conclude that higher arterial pressure in pediatric children with hydronephrosis cannot be explained by preoperative stress. Importantly, the reduction of arterial pressure in young children with hydronephrosis occurred even though 6 months had passed since the surgery, and one would expect the arterial pressure to increase slightly with increased age [27].

Oxidative stress is characterized by the increased production of reactive oxygen species (ROS) and reduced antioxidant capacity. This oxidative stress is prevalent in patients with kidney disease and has been suggested as an important pathogenic mechanism associated with an increased risk of cardiovascular disease [28–30]. Several experimental studies have shown that also ureteral obstruction-induced injuries in mice are associated with oxidative stress [31–34]. In our previous studies, we demonstrated that hydronephrosis, induced by partial unilateral obstruction, leads to the development of hypertension in both rats and mice [9, 10]. Underlying pathological mechanisms in this model include altered prostaglandin and thromboxane signaling [35, 36] in the diseased kidney together with oxidative stress and reduced NO bioavailability [12–15]. To the best of our knowledge, the above-mentioned pathological mechanisms have never been investigated in patients with hydronephrosis.

The measurement of oxidative stress in body fluids is complicated because of the very short half-life of ROS. Isoprostanes, which are mainly formed through free-radical catalyzation of arachidonic acid, are now considered to be reliable biomarkers of oxidative stress [37]. Here we show that pathways associated with oxidative stress due to impaired prostaglandin and thromboxane signaling [38, 39], as well as biomarkers of oxidative stress and lipid peroxidation (F_2_-isoprostanes) [37, 40], were significantly increased in patients with hydronephrosis and, importantly, they were all reduced following surgery.

Oxidative stress due to the increased generation of ROS from vascular NADPH oxidase and mitochondria is often associated with reduced NO levels due to scavenging (i.e., reaction with superoxide leading to peroxynitrite formation) [41]. However, in contrast to the results obtained in our previous experimental studies using adult rodents, we did not observe any signs of reduced NO bioavailability or signaling in young patients with hydronephrosis. Actually, NOS activity appeared to be increased before surgery and normalized following management of the obstruction. This finding warrants future investigations, but we speculate that in patients characterized with oxidative stress an upregulation of enzymatic systems that generate NO (including NOS) may partially maintain vascular NO homeostasis despite the increased generation of ROS. This study only included children, but it is possible that this compensation (upregulation) of the NO generating system due to oxidative stress may become exhausted over time and hence lead to progressively increased arterial pressure with a higher risk of developing renal injuries. If this hypothesis of imbalance between oxidant and antioxidant systems hold true, as described in patients with chronic kidney disease [28–30], surgical management of hydronephrosis would be suggested in all cases, even if the arterial pressure is normal and the patient has no other symptoms/diagnosis.

In conclusion, this novel prospective study demonstrates significantly higher arterial pressure and oxidative stress in children with hydronephrosis compared with healthy controls. The elevated arterial pressure can be normalized by surgical correction of the obstruction, which supports our previous experimental and clinical case report studies. Currently, normalized reference values of 24-h ambulatory blood pressure in very young children are limited [27, 42, 43]. Once reference data in this young age group become available, together with additional data on arterial pressure in pediatric patients with hydronephrosis, we hope that cutoff values can be defined and that these will assist decision-making regarding surgical management of hydronephrosis to avoid the adverse effects of high arterial pressure in the future.
